# MISTIC: A prediction tool to reveal disease-relevant deleterious missense variants

**DOI:** 10.1371/journal.pone.0236962

**Published:** 2020-07-31

**Authors:** Kirsley Chennen, Thomas Weber, Xavière Lornage, Arnaud Kress, Johann Böhm, Julie Thompson, Jocelyn Laporte, Olivier Poch

**Affiliations:** 1 Complex Systems and Translational Bioinformatics (CSTB), ICube laboratory – CNRS, Fédération de Médecine Translationnelle de Strasbourg (FMTS), University of Strasbourg, Strasbourg, France; 2 Institut de Génétique et de Biologie Moléculaire et Cellulaire, INSERM U1258, CNRS UMR7104, University of Strasbourg, Illkirch, France; Johannes Gutenberg Universitat Mainz, GERMANY

## Abstract

The diffusion of next-generation sequencing technologies has revolutionized research and diagnosis in the field of rare Mendelian disorders, notably *via* whole-exome sequencing (WES). However, one of the main issues hampering achievement of a diagnosis *via* WES analyses is the extended list of variants of unknown significance (VUS), mostly composed of missense variants. Hence, improved solutions are needed to address the challenges of identifying potentially deleterious variants and ranking them in a prioritized short list. We present MISTIC (MISsense deleTeriousness predICtor), a new prediction tool based on an original combination of two complementary machine learning algorithms using a soft voting system that integrates 113 missense features, ranging from multi-ethnic minor allele frequencies and evolutionary conservation, to physiochemical and biochemical properties of amino acids. Our approach also uses training sets with a wide spectrum of variant profiles, including both high-confidence positive (deleterious) and negative (benign) variants. Compared to recent state-of-the-art prediction tools in various benchmark tests and independent evaluation scenarios, MISTIC exhibits the best and most consistent performance, notably with the highest AUC value (> 0.95). Importantly, MISTIC maintains its high performance in the specific case of discriminating deleterious variants from benign variants that are rare or population-specific. In a clinical context, MISTIC drastically reduces the list of VUS (<30%) and significantly improves the ranking of “causative” deleterious variants. Pre-computed MISTIC scores for all possible human missense variants are available at http://lbgi.fr/mistic.

## Introduction

Next-Generation Sequencing technologies, such as Whole Exome Sequencing (WES) involving the targeted sequencing of exonic regions of all known protein-coding genes, have gradually replaced conventional approaches for the study of rare Mendelian disorders since 2010 [1]. Their usage is shifting from research investigations of disease-causing variants to routine clinical exome analysis for diagnosis of Mendelian disorders with known genetic aetiology [2, 3]. However, with a diagnostic rate of ~40% for exome analyses, the identification of the deleterious variants, even in the coding regions, remains laborious [4–6]. The unsolved exomes usually result in extensive lists of variants, including numerous Variants of Unknown clinical Significance (VUS). The VUS are variants for which the pathogenicity (either benign or deleterious) could not be reliably determined given all available evidence (databases, collections of exomes etc), according to recommendation criteria from scientific communities, such as the Association for Molecular Pathology (AMP) [7] or the American College of Medical Genetics (ACMG) [8]. The VUS are mainly composed of missense variants, which make up to ~60% of ‘Uncertain significance’ variants in the ClinVar database [9].

The AMP and ACMG guidelines provide several criteria to classify deleterious/benign variants, in order to filter, prioritize or reduce the list of VUS into a shorter list of candidate variants that is amenable for expert review and additional experimental validation [10, 11]. For example, the minor allele frequency (MAF) (*e*.*g*. criteria PM2, BS1, BA1 of the ACMG) representing the observed frequency of a given variant in control healthy cohorts, has been demonstrated to be a very powerful filter. However, MAF values are often missing for deleterious or population-specific variants. To facilitate the evaluation of missense variants effects, several deleteriousness prediction tools have been developed that integrate a number of additional criteria [8, 12], such as the impact of the variant on the protein structure and/or function, the evolutionary conservation, or the physiochemical and biochemical properties of amino acids (*e*.*g*. SIFT [13], PolyPhen2 [14], VEST4 [15]). These tools have an accuracy ranging from 65 to 80% when benchmarked on known disease missense variants [16, 17]. Since individual tools tend to disagree on some missense variants, a novel type of ensemble prediction tools has recently emerged (*e*.*g*. Condel [18], CADD [19], MetaLR/MetaSVM [20], FATHMM-XF [21], Eigen [22], REVEL [23], M-CAP [24], ClinPred [25], and Primate AI [26]). The ensemble prediction tools combine the power of individual tools in order to achieve higher classification accuracies up to ~90% [17]. Nevertheless, the tools can still produce ambiguous predictions or even no prediction at all for some missense variants, contributing to the extended list of VUS (criteria PP3 of the ACMG) with a poor ranking of causative variants.

Here, we present MISTIC (MISsense deleTeriousness predICtor), a new supervised machine-learning model dedicated to the prediction of deleterious missense variants. MISTIC integrates a Soft Voting system [27] based on two optimized complementary machine-learning algorithms (Random Forest [28] and Logistic Regression [29]). The algorithms were trained to distinguish deleterious from benign missense variants based on a selection of 113 missense features, ranging from multi-ethnic MAF and evolutionary conservation constraints, to changes in amino acid physiochemical and biochemical properties. The performance of MISTIC is compared to other recent state-of-the-art prediction tools (Eigen, FATHMM-XF, REVEL, M-CAP, ClinPred and PrimateAI) in a series of benchmark tests designed to represent different variant analysis scenarios. We show that MISTIC has the best performance in predicting and ranking deleterious missense variants in coding regions. Moreover, in a clinical usage context, we demonstrate that MISTIC drastically reduces the list of VUS, and improves the ranking of the “causative” deleterious variants. To make MISTIC easily usable and accessible for future developments, we provide pre-computed scores for all possible human missense variants.

## Materials and methods

### Features

To describe missense variants, 714 features in 4 main categories were initially collected (S1 Table):

1. 8 multi-ethnic MAF [30]: all exomes (global MAF), African/African-American (AFR), Latino American (AMR), Ashkenazi Jewish (ASJ), East Asian (EAS), Finnish (FIN), Non-Finnish European (NFE), South Asian (SAS).
2. 8 conservation measures: PhastCons (primates, mammals, vertebrates) [31], PhyloP (primates, mammals, vertebrates) [32], SiPhy [33], GERP++ [32].
3. 690 functional measures: constrained coding regions (CCRs) [34], Missense badness, PolyPhen-2, and Constraint score (MPC) [35], physicochemical and biochemical properties from the AAindex databases (amino acid features) [36].
4. 7 pathogenicity predictors based on deleteriousness scores from different prediction tools: SIFT, PolyPhen2, VEST4, Condel, CADD, MetaSVM and MetaLR.

The features for the missense variants are based on the GRCh37 genome assembly and were extracted using Variant Ensembl Predictor (VEP) v96 [37] and VCFAnno v0.3.1 [38] from the CADD v1.4 and dbNSFP v4.0b2 [39] databases.

### Training and test sets

MISTIC was trained and tested using variants from the VarData set, which is composed of (i) a positive set corresponding to rare deleterious missense variants, and (ii) a negative set corresponding to rare benign missense variants (S2 Table).

For the positive set, 38,565 deleterious missense variants with a “Pathogenic” clinical significance interpretation (CLNSIG) were selected from the ClinVar [9] VCF file (release of 30/09/2018). This list of variants was further filtered to select only 15,219 high confidence variants with a review status (CLNREVSTAT) “criteria provided” by the submitter, provided by “multiple submitters”, a “reviewed by expert panel” or using “practice guideline”, and “no conflicts” among the multiple interpretations submitted. Additionally, from the curated HGMD Pro [40] VCF file (version 2018.1), 76,523 missense variants with “Disease-Mutation” (DM) STATUS tag were selected as high-confidence deleterious missense variants. The resulting lists of variants from both ClinVar and HGMD Pro were then filtered to exclude:

1. any overlapping variants with the training set of the 7 prediction tools (PolyPhen-2 HUMVAR & PolyPhen-2 HUMDIV, SIFT, VEST4, Condel, CADD, MetaLR, MetaSVM) used as features in MISTIC to prevent type 1 circularity errors [17],
2. variants without a full annotation coverage of the features used in MISTIC (see Model definition section).

Finally, the VarData positive set contains 11,190 high confidence deleterious missense variants after merging the non-filtered variants from ClinVar and HGMD Pro databases.

For the negative set, rare benign missense variants were obtained from the gnomAD database, which combines variation data from over 125,000 exomes and over 15,000 genomes. Since no individuals in this database have any of the known severe childhood Mendelian disorders, it is assumed that highly penetrant disease-causing missense variants will be rare in this database (MAF < 1%). The missense variants with a depth coverage >30X, were filtered to exclude (i) any overlapping variants in ClinVar and HGMD Pro databases, (ii) type I circularity error variants and (iii) variants without a full annotation coverage of MISTIC features, which resulted in 5,599,566 variants. The resulting list was divided into two sets: (i) Benign_VarData set comprising 11,190 randomly selected variants to match the size of the positive set for the training and testing of MISTIC, and (ii) Benign_EvalSet that contains the rest of the variants and serves as a negative set for the further evaluation of MISTIC (see below).

In order to train the supervised machine-learning models in MISTIC, 10,070 variants (~90% of VarData) were used from both positive and negative sets (denoted VarTrain). The remaining 992 variants (~10% left of VarData) in both positive and negative sets (denoted VarTest) were then used to test the performance of MISTIC.

### Evaluation scenarios

To further evaluate the performance of MISTIC compared to other prediction tools, we collected six additional sets, including (i) two sets of deleterious variants, (ii) a set of rare benign variants and (iii) three population-specific variants (S3 Table).

1. Del_EvalSet contains two sets of deleterious variants:
  The ClinVarNew set was generated to assess the ability of the different tools to predict novel deleterious variants. We therefore identified recent deleterious missense variants present in the ClinVar database of April 2019 (release of 2019/04/03) and absent from the version of September 2018 (release of 2018/09/30 used to construct the VarTrain set). After applying the same filters for high confidence deleterious missense variants as described above for ClinVar in the VarData positive set, 437 “novel” high confidence deleterious missense variants were obtained. To avoid circularity errors, the variants overlapping with the training sets of the tools used in the benchmark study (PolyPhen-2, SIFT, VEST4, Condel, CADD, MetaLR, MetaSVM, VarTrain) were removed (referred to later as circularity error filter). After applying the circularity error filter, 388 variants were obtained. However, ClinPred and M-CAP did not provide any scores for 101 variants, so for fair comparison only the resulting 287 deleterious missense variants were used in the benchmark test.
  The DoCM set was generated by selecting deleterious missense variants from the Database of Curated Mutations (version 3.2), derived from the literature and composed of curated mutations observed in cancer [41]. The circularity error filter was applied to the initial 226 pathological missense variants and variants overlapping with the ClinVarNew set were removed, resulting in 126 deleterious missense variants.
2. Benign_EvalSet contains one set of benign variants with MAF data.
  The Benign_EvalSet was constructed to evaluate the ability of the tools to predict rare benign variants with different levels of MAF. As described above, the Benign_EvalSet comprises 4,974,224 missense variants, after applying the circularity error filter and removing the variants used in VarTrain and VarTest.
3. PopSpe_EvalSet contains thee sets of population-specific variants, for which no MAF information is available.
  The UK10K set was constructed by selecting population-specific variants present in 3,781 healthy individuals from two British cohorts of European ancestry present in the UK10K project [42], namely the Avon Longitudinal Study of Parents and Children (ALSPAC) [43] and TwinsUK [44]. Different filters were applied to the initial 295,218 missense variants: (i) a depth coverage >30X, (ii) the circularity error filter, (iii) the population specific filter, which removes variants with MAF data or present in the VarData set, evaluation sets (ClinVarNew, DoCM), or in the other population sets. Finally, 34,973 UK10K-population-specific variants were obtained.
  The SweGen set was constructed by selecting population-specific variants present in 1,000 healthy Swedish individuals from the SweGen project [45]. After applying the same filters as for UK10K, 25,635 SweGen-population-specific variants were obtained.
  The WesternAsia set was constructed by pooling variant sets from 269 healthy Kuwaiti natives (comprising 109 individuals of Saudi Arabian tribe ancestry, 126 individuals of Persian ancestry, 34 individuals of Bedouin ancestry) [46] and 16 healthy Turkish individuals [47]. After applying the same filters as for UK10K, 14,594 WesternAsia-population-specific variants were obtained.

Different combinations of the six evaluation sets were then used to construct three prediction scenarios:

1. ClinVarNew and Benign_EvalSet, to distinguish novel deleterious variants from rare benign variants,
2. DoCM and Benign_EvalSet, to distinguish known deleterious variants from rare benign variants,
3. ClinVarNew/DoCM and PopSpe_EvalSet. to identify deleterious variants in population-specific datasets without MAF data.

### Clinical context scenarios

We constructed different datasets representing both simulated and real disease exomes.

The 1KG set comprises simulated disease exomes, in which we introduce a randomly selected deleterious missense variant (from one of the deleterious sets described above) into the 1092 individual background exomes from the 1000 Genomes Project [48]. The simulated disease exomes were then annotated using VEP and VCFAnno. The variants were filtered according to community best practices, such as depth coverage >10X and MAF <1% in control healthy population databases. After applying the circularity filter, there was an average of 420 missense variants per simulated disease exome.

The MyoCapture set represents more than 1,200 clinical exomes from the French MyoCapture consortium on congenital myopathies [49]. The 15 selected resolved cases correspond to recently identified disease-causing deleterious variations, published after 2016 and not included in VarTrain. These cases were considered as solved if: (i) the disease-causing deleterious variant is in a known myopathy-causative gene and the gene associated phenotypes clinically match the patient’s phenotypes; (ii) the disease-causing deleterious variant is in a novel disease gene with strong genetic validation (*e*.*g*. segregation analysis, multiple families with variants in the same gene, similar phenotype) and functional evidence (*e*.*g*. animal models reproducing the patient phenotypes) according to the ACMG’s recommendations. The sequencing reads were mapped to the GRCh37/hg19 assembly of the human genome using BWA-MEM v0.7.10-r789 [50]. Variants were called using GATK v4.0.3.0 following the Haplotype Caller workflow from GATK best practices [51]. The procedures for the annotation and filtering steps, described above for the 1KG set were also applied here. After applying the circularity filter, there was an average of 1,566 missense variants per clinical exome.

### Model definition

Using the python scikit-learn library v0.20.2, we trained Random Forest [28] and Logistic Regression [29] machine learning algorithms on the VarTrain missense variants, which includes 10,070 deleterious variants as the positive set and 10,070 benign variants as the negative set. The design of MISTIC was done in three main steps. First, a selection and implementation of the most informative variant features (detailed above) for each algorithm, the Recursive Feature Elimination method (RFE) was used [52]. RFE is a method that enables machine learning algorithms to perform feature selection by iteratively training a model, ranking features (by assigned weights or coefficients), and then removing the lowest ranking features. Second, the predictions of the Random Forest and the Logistic Regression algorithms were then integrated in a Soft Voting system. In contrast to classical majority voting (Hard Voting), a Soft Voting system calculates the weighted average probabilities. Third, the optimized combination of parameters for the Random Forest and Logistic Regression algorithms and the hyper-parameters of their relative weights in the Soft Voting system was obtained after a grid search optimization of 20 iterations with 5 cross-validations each time.

The score generated by the Soft Voting system ranges from 0 to 1 and represents the probability of a given missense variant to be classified as deleterious. By default, missense variants with scores >0.5 are classified as deleterious and missense variants with scores <0.5 are classified as benign.

### Benchmarking statistics

The performance of MISTIC was compared to six recent state-of-the-art tools for prediction of deleterious variants: Eigen, PrimateAI, FATHMM-XF, REVEL, M-CAP and ClinPred. However, since the deleteriousness scores from these tools were not always available for every missense variant (ranging from 3.6% of the missense variants for REVEL up to 9.4% for M-CAP), we excluded variants without scores. The thresholds recommended by the authors (S4 Table) were used to compare the prediction performance of the different tools on the evaluation sets. Furthermore, for clinically relevant applications, the prediction and ranking performances were compared on sets corresponding to simulated disease exomes (1KG) and real clinical exomes (MyoCapture).

To compare the performance of the prediction tools, we used several statistical metrics derived from a confusion matrix. To achieve this, we identified a correctly classified variant as a true positive (TP) if and only if the variant corresponded to the positive class (deleterious) and as a true negative (TN) if and only if the variant corresponded to the negative class (benign). Accordingly, a false positive (FP) is a negative variant (benign) that is classified as positive (deleterious) and a false negative (FN) is a positive variant (deleterious) classified as a negative one (benign). From these different classification statistics, we calculated 12 performance metrics (S5 Table) as described in the Human Mutation guidelines [53], notably:

1. Sensitivity—proportion of identified true deleterious variants compared to all the true deleterious variants.
2. Specificity—proportion of identified true benign variants compared to all the true benign variants.
3. Precision–proportion of identified true positive deleterious variants over all variants predicted as deleterious.
4. Area under the Receiver Operating Characteristics (ROC) curve (AUC). The AUC can take values between 0 and 1. A perfect tool has an AUC of 1 and the AUC of a random tool is 0.5.
5. F1 score—measure of prediction accuracy, with a balanced use of precision and sensitivity. The higher the F1 score, the higher the accuracy of the tool.
6. Matthews Correlation Coefficient (MCC)—considers true and false positives and negatives to represent the degree of correlation (range from -1 to 1) between the observed and predicted binary classifications. The MCC is generally regarded as a balanced method to evaluate tools. An MCC of -1 indicates a completely wrong binary tool, while an MCC of 1 indicates a completely correct binary tool.
7. Log Loss value—measures the divergence of a tool from the true variant labels (true deleterious or true benign), *i*.*e*. it measures the associated degree of uncertainty for a tool. The Log Loss value ranges from +∞ to 0. In this case, a good tool will have a low Log Loss value, hence a low degree of uncertainty in its predictions.
8. Diagnostic Odd Ratio (DOR)–measures the effectiveness of a diagnostic binary classification test. It is defined as the ratio of the odds of the test being positive if the variant is deleterious relative to the odds of the test being positive if the variant is benign. The DOR value ranges from zero to +∞ and hence higher DOR are indicative of better tool performance.

## Results

### Variant prediction model

In order to accurately classify deleterious and benign missense variants, we built the MISTIC model based on a Soft Voting system that combines predictions from Random Forest and Logistic Regression machine learning algorithms. We initially defined 714 features to fully characterize the missense variants (VarTrain dataset) used to train the model (S1 Table). However, a common problem of such high-dimensional data sets is the presence of correlated predictors, which impacts the ability of the algorithms to identify the strongest predictors. Hence, to reduce the dimensionality of our data, we identified the most important features for each of the Random Forest and Logistic Regression algorithms independently, using the RFE method.

The data in S1 Fig show that the performance of the Random Forest models increases as the number of features decreases, ranging from an AUC value of 0.852 for a model with 714 features to a peak AUC value of 0.895 for a model with 10 features. In contrast, the performance of the Logistic Regression models with less than 113 features are lower with a mean AUC value of 0.820, while models with more than 113 features have a stable performance with a mean AUC value of 0.826. Since the Soft Voting system requires that both algorithms have the same number of features, a cutoff was defined at 113 features for an optimised performance combining both algorithms. The 113 selected features cover 3 main categories: (i) multi-ethnic MAF values (6 features), (ii) functional and conservation measures (100 features), and (iii) scores from missense prediction tools (7 features) (Table 1, see detailed list in S6 Table).

**Table 1:** Selected features included in the Soft Voting system of MISTIC.

Category	Name	Number of features	Description
**Minor Allele Frequencies**	minor allele frequencies	6	minor allele frequencies for 6 populations: all exomes (global MAF), African (AFR), American (AMR), East Asian (EAS), None Finnish European (NFE), South Asian (SAS)
**Conservation measures**	PhastCons	3	phastCons conservation score based on three categories of multiple alignments: (i) 100 vertebrate genomes, (ii) 30 mammalians, and (iii) 17 primates. The larger the score, the more conserved the site
	PhyloP	3	phyloP (phylogenetic p-values) conservation score based on three categories of multiple alignments: (i) 100 vertebrate genomes, (ii) 30 mammalians, and (iii) 17 primates. The larger the score. the more conserved the site
	SiPhy 29way logOdds	1	The estimated stationary distribution of A, C, G and T at the locus using SiPhy algorithm based on 29 mammalian genomes
	GERP++_RS	1	Identified constrained elements in multiple alignments
**Functional measures**	CCRS	1	The score reflects the intolerance of constrained coding regions of protein-coding genes for protein-altering variants
	MPC	1	A deleteriousness prediction score for missense variants based on regional missense constraints.
	AAindex	90	The AAindex substitution matrices for different physicochemical and biochemical properties of amino acids.
**Pathogenicity predictors**	SIFT	1	Prediction of the impact of an amino acid substitution on the protein function
	PolyPhen 2	1	Prediction of the impact of an amino acid substitution on the structure and function of a protein using straightforward physical and comparative considerations
	VEST4_score	1	Machine learning method predicting the functional significance of missense mutations based on the probability that they are pathogenic
	Condel	1	Weighted average of the normalized scores of five methods (SIFT, PolyPhen2, Logre, MAPP, MutationAssessor)
	CADD PHRED	1	Machine learning scoring model that integrates more than sixty annotation features into a single metric, to distinguish variants that survived naturel selection from simulated mutations
	MetaLR	1	Logistic Regression model combining multiple variant scoring metrics
	MetaSVM	1	Support Vector Machine model combining multiple variant scoring metrics

Finally, the MISTIC model was trained on the VarTrain set, using the 113 selected features. Twenty iterations on a randomized grid search and a 5 cross-validation on VarTrain were used to obtain the hyper-parameters for the most optimized combination of the Random Forest and the Logistic Regression algorithms (S7 Table). Each algorithm calculated different weights for the individual features (See S2 Fig and S8 Table). For the Random Forest, the 5 most predominant features are the global MAF (19.73%), MetaSVM (9.44%), MetaLR (6.93%), VEST4 (5.84%) and Condel (5.39%). For the Logistic Regression, the 5 strongest features are VEST4 (16.16%), MetaLR (8.63%), MetaSVM (5.00%), PolyPhen (3.88%) and the AAindex matrix MIYS930101 [54] (3.22%) that evaluates contact frequencies in protein structures for all residues.

### Comparison of MISTIC with individual component features and other prediction tools on VarTest set

**Fig 1:**
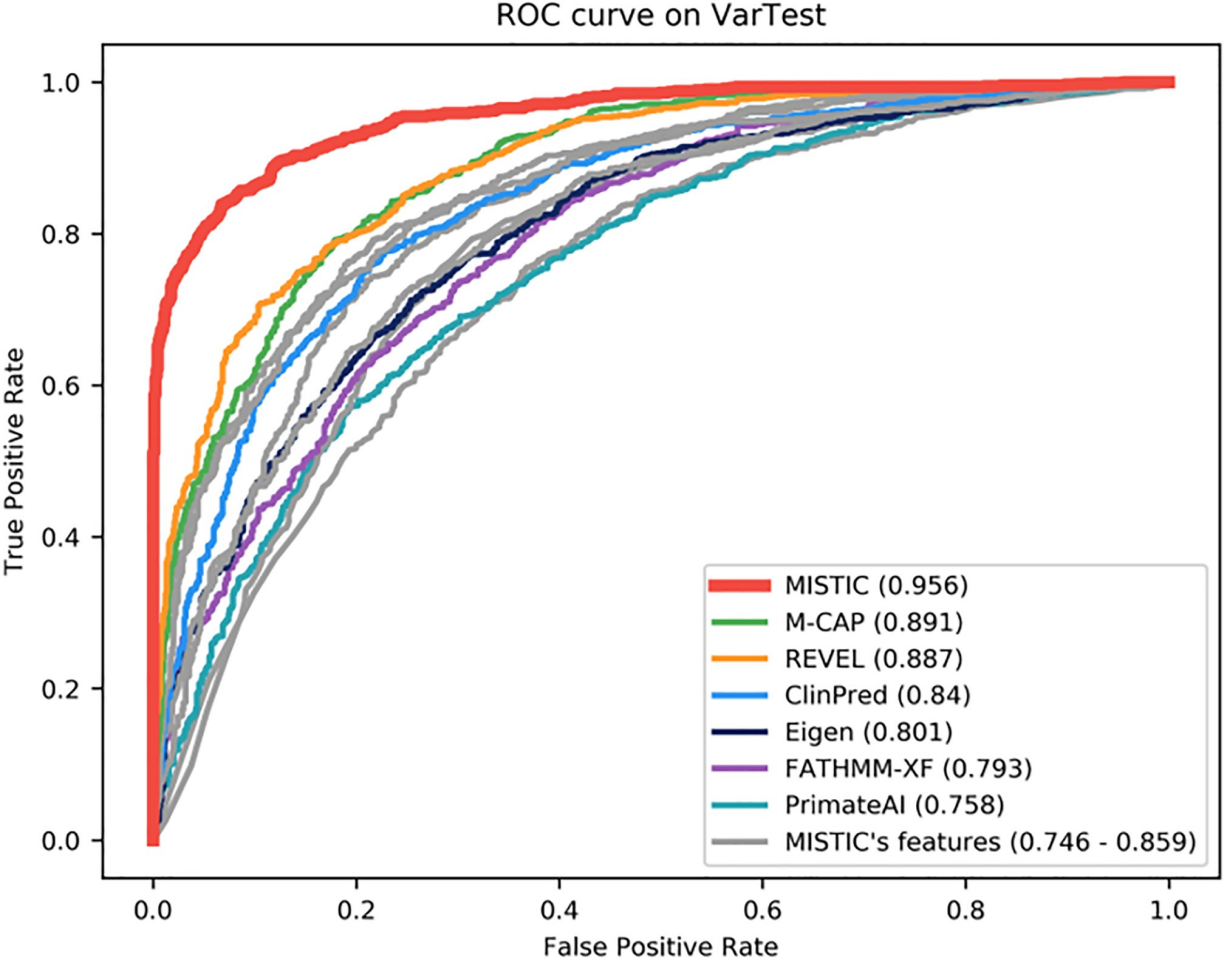
Performance of missense prediction tools on VarTest set. MISTIC was compared to individual component features (MetaSVM, MetaLR, VEST4, Condel, CADD, PolyPhen2, SIFT) used in its model (in grey) and the best-performing tools recently published (in color). The Area Under the receiver operating characteristics Curve (AUC) is shown in brackets.

The performance of the MISTIC Soft Voting system was compared with other prediction tools using the VarTest set. As might be expected, MISTIC globally outperforms each of its individual component features (MetaSVM, MetaLR, VEST4, Condel, CADD, PolyPhen2, SIFT). However, MISTIC also performs better than the state-of-the-art missense prediction tools (Eigen, PrimateAI, FATHMM-XF, REVEL, M-CAP and ClinPred) with the highest AUC value of 0.956 (S9 Table, Fig 1). M-CAP has the second-best overall performance, with an AUC value of 0.891. M-CAP has the highest sensitivity of 0.955, but this comes at the cost of a low specificity value of 0.547. In contrast, MISTIC has a balanced sensitivity of 0.863 and specificity of 0.901. Among the individual component features of MISTIC, the MetaLR score has the best performance with an AUC value of 0.859. We also calculated other metrics, such as the F1 score (measures accuracy based on the balance between precision and sensitivity), the Log Loss value (measures the degree of uncertainty associated with a prediction) and the Diagnostic Odds Ratio (measures the effectiveness of a deleterious prediction relative to the odds of a deleterious variant and the odds of a benign variant). Here, MISTIC has the highest F1-score of 0.881, the highest DOR value of 57.347, as well as the lowest Log Loss value of 4.082.

### Evaluation of MISTIC in different variant analysis scenarios

The generalizability and relevance of MISTIC’s prediction performance was further compared to the other prediction tools using datasets representing different scenarios. It is important to note that the variant sets used in these scenarios are independent from the variant sets used for the model training (VarTrain) and initial testing (VarTest) described in the previous section.

First, we tested the ability of the prediction tools to differentiate novel deleterious variants (ClinVarNew set) or known deleterious variants from diverse sources (DoCM set), from rare benign variants at 5 MAF levels (<0.01, <0.005, <0.001, <0.0001, singleton in Benign_EvalSet). Since each MAF set does not have the same number of deleterious variants, the corresponding number of benign variants was randomly selected to obtain balanced pairs of deleterious-benign evaluation sets. This procedure was repeated 10 times and at each iteration, a different random set of benign variants was used.

**Fig 2:**
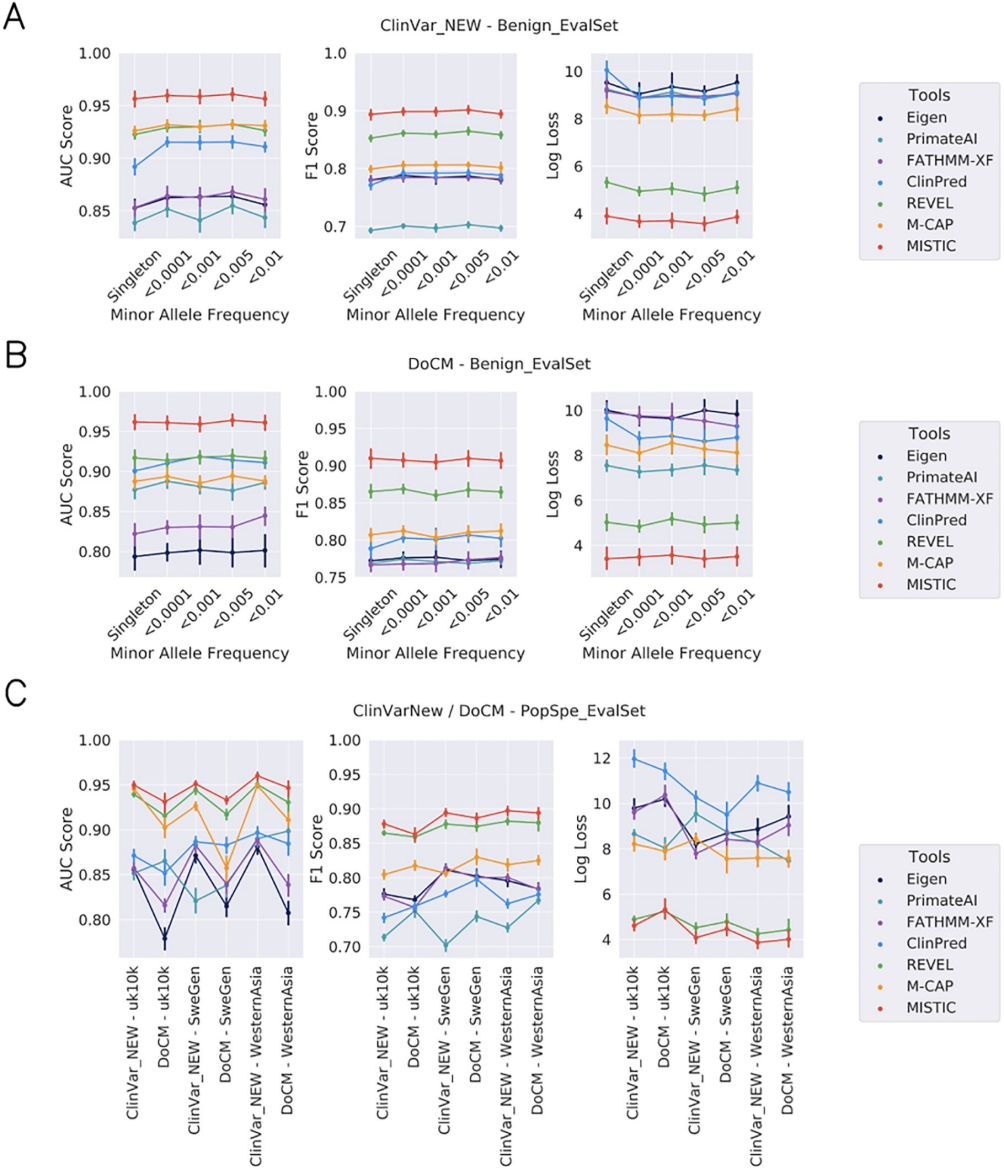
Evaluation of prediction tools on different variant analysis scenarios. The performance of MISTIC was compared to other missense prediction tools for the discrimination of deleterious variants from rare benign variants and population-specific missense variants. All prediction tools were evaluated using novel deleterious variants (Fig 2A - ClinVarNew and Benign_EvalSet set), known deleterious variants from diverse sources (Fig 2B - DoCM and Benign_EvalSet set), rare benign variants with MAF data (<0.01, <0.005, <0.001, <0.0001, singleton) or benign variants without MAF (ClinVarNew/DoCM and PopSpe_EvalSet: UK10K, SweGen, WesternAsia; Fig 2C).

Overall, MISTIC has the most consistent and best performance in discriminating deleterious variants from rare benign variants, with the highest mean AUC value on all the different scenarios (Fig 2). For the scenario involving novel deleterious variants (ClinVarNew set; Fig 2A, S10 Table) and rare benign variants, MISTIC has the highest mean AUC value of 0.963 ± 0.002, mean F1 score of 0.907 ± 0.002, mean DOR value of 92.548 ± 5.182, and the lowest mean Log Loss value of 3.332 ± 0.099. In terms of mean AUC and mean DOR values, M-CAP is the second best-performing tool with a mean AUC value of 0.930 ± 0.002 and a mean DOR value of 39.516 ± 1.650. However, in terms of mean F1 score, REVEL is the second-best performing tool (0.859 ± 0.004), as well as in terms of mean Log Loss value (5.048 ± 0.186).

For the scenario involving known deleterious variants from diverse sources (DoCM; Fig 2B, S11 Table) and rare benign variants, the same tendency was observed. Here, MISTIC has the best performance, with the highest mean AUC value of 0.968 ± 0.001, mean F1 score of 0.920 ± 0.003, mean DOR value of 125.642 ± 6.905, and the lowest mean Log Loss value of 2.981 ± 0.099.

Since the global MAF is an important feature in the MISTIC model (see S2 Fig), although MAF values are often missing for deleterious and population-specific benign variants, we evaluated the performance of MISTIC in discriminating deleterious variants from rare benign variants when no MAF data are available. To do this, benign population-specific variants were collected from three different populations, namely UK10K, SweGen and WesternAsia. For each deleterious set (ClinVarNew, DoCM), the corresponding number of benign variants was randomly selected from each population-specific set of variants. The deleterious and benign variants were scored by MISTIC and the six other missense prediction tools. This procedure was repeated 10 times, with a different random selection of the benign variants each time. For the six different combinations of two deleterious sets (ClinVarNew, DoCM) and three benign population-specific sets (UK10K, SweGen, WesternAsia), MISTIC has the best performance for three of them, with the highest mean AUC value of 0.945 ± 0.009 (Fig 2C, S12 Table). The second best-performing prediction tool is REVEL, with an overall mean AUC value of mean of 0.933 ± 0.012, F1 score 0.873 ± 0.007 and a mean Log Loss value of 4.688 ± 0.287. ClinPred has the highest DOR values for three of the combinations of variant sets and MISTIC has the highest DOR values for the other combinations. ClinPred has the highest sensitity (1) and DOR value (∞) in the combinations of variants based on the known deleterious variants (DoCM set). This is probably due to an overlap between the ClinPred training set and the DoCM set, leading to a problem of overfitting.

### Performance on simulated disease exomes

In the context of a typical Mendelian disease exome analysis, even after most common benign variants have been removed with a standard allele frequency filter (MAF >1%), the challenge is to identify one or two rare causative deleterious variants among hundreds of predicted deleterious variants. Indeed, with current limited resources (time and cost), it not feasible to experimentally validate large numbers of candidate variants. To evaluate the ability of the prediction tools to prioritize the causative variants, we simulated Mendelian disease exomes by introducing one “causative” deleterious variant (from Del_EvalSet) in the background exomes of healthy individuals from the 1000 Genomes Project. The simulated disease exomes thus contained one “causative” variant and an average of ~420 missense variants per exome (see section Materials and methods).

**Fig 3:**
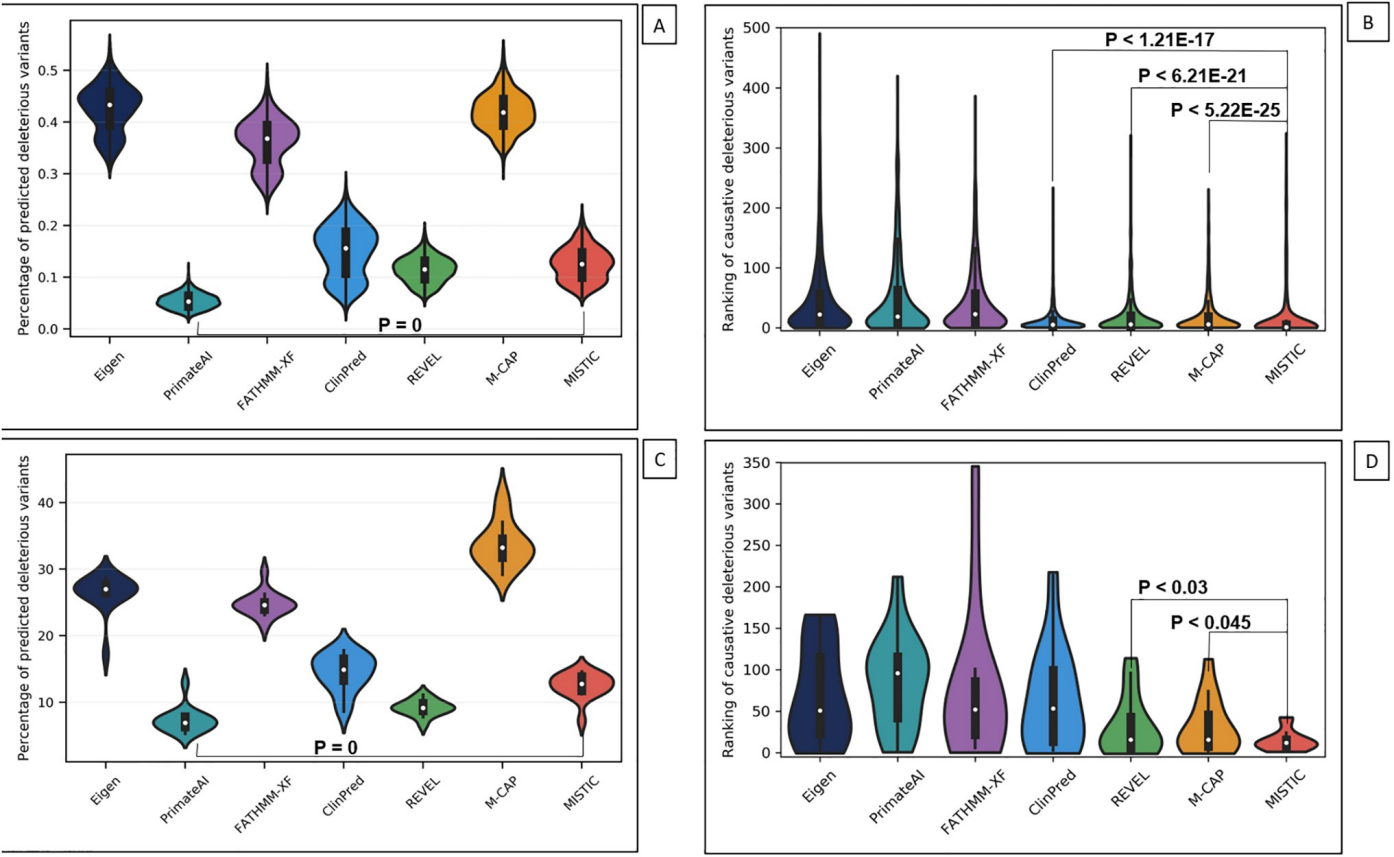
Evaluation of the different missense prediction tools using simulated and real disease exomes. A–Distribution of the percentage of predicted deleterious variants in the simulated disease exomes. B–Ranking of the “causative” deleterious variants introduced in simulated disease exomes. C–Distribution of the percentage of predicted deleterious variants on the exomes of the MyoCapture project. D–Ranking of the causative deleterious variants identified in real congenital myopathy exomes from the MyoCapture project.

First, we calculated the percentage of predicted deleterious variants obtained by the different tools, again using the authors’ recommended threshold each time. The objective is to have the “causative” variants among the shortest list of predicted deleterious variants, that is trackable for a manual expert review. PrimateAI generated the shortest the list of variants by predicting only 5.393 ± 1.463% of the 1KG exomes variants as deleterious, while MISTIC’s prediction was of 12.529 ± 3.195% (Fig 3A and S13 Table). Next, we evaluated the ability of the prediction tools to rank the “causative” variants among the top-scoring deleterious variants. We calculated the mean ranks of the “causative” variants introduced in the disease exomes after sorting the scores for each prediction tool (Fig 3B, and S13 Table). Overall, MISTIC has the best performance with a median rank value of 2, (mean rank: 14.092 ± 34.968) for the “causative” variants. The performance of MISTIC is significantly higher (Mann-Whitney P < 1.21 x 10^−17^) than the second-best tool, ClinPred, which has a median rank of 5 (mean rank: 11.155 ± 19.760).

### Performance on real clinical cases from a myopathy cohort

Finally, to represent a clinical practice scenario, we compared the performance of MISTIC to Eigen, PrimateAI, FATHMM-XF, ClinPred, M-CAP and REVEL, using 15 recently solved clinical exomes from the French Myocapture cohort on congenital myopathies. After applying the best-practice filtering procedures (See Material and Methods), the 1566 missense variants per exome were scored by the prediction tools (S14 Table).

As for the simulated disease exomes, PrimateAI achieves the largest reduction of the list of predicted deleterious variants (92.14% ± 2.19%), while MISTIC is the third best method (82.68% ± 2.36%) (Fig 3C, and S14 Table). However, in terms of ranking of the causative variants, MISTIC has the best performance with a median rank of 12 (mean: 14.67 ± 12.35) in the Myocapture exomes. M-CAP and REVEL were performed second-best with a median rank of 16 (M-CAP mean rank: 30.93 ± 32.59; REVEL mean rank: 31.07 ± 35.530). The ranking performance of MISTIC is significantly different from M-CAP and REVEL (P < 0.045 and P < 0.030 respectively).

### Comparison of scores for deleterious and benign variants

**Fig 4:**
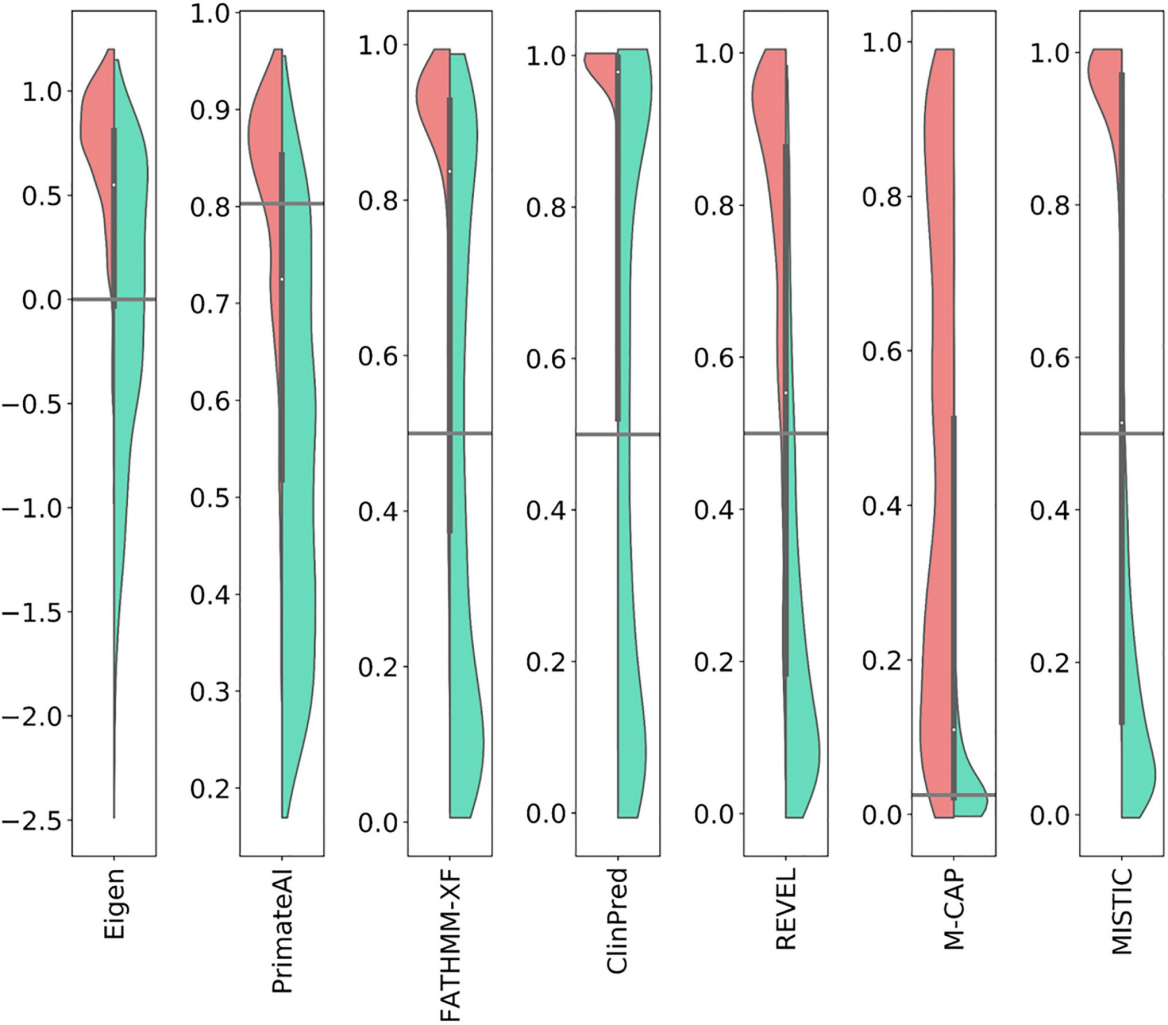
Distribution of scores for deleterious and benign variants. The variants of the deleterious (Del_EvalSet) and benign sets with MAF (Benign_EvalSet) were pooled and the distribution of the scores for deleterious and benign variants were represented using violin plots. Red area–distribution of scores for deleterious variants. Green area–distribution of scores for benign variants. Black line–recommended threshold.

To better understand the prediction behavior of MISTIC and the other tools, the score distribution of all variants in the pooled deleterious (Del_EvalSet) and benign sets (Benign_EvalSet, PopSpe_EvalSet) was visualized using violin plots (Fig 4, S3 Fig). Each tool provides a score and an associated class (deleterious or benign) based on the recommended threshold given by the authors (S4 Table). We therefore analyzed the score distributions for deleterious variants and benign variants with a MAF (Fig 4), and observed that tools (Eigen, PrimateAI, FATHMM-XF, ClinPred) that did not perform well (DOR value < 10) in our evaluation experiments generally have a poor performance in classifying benign variants. Around 50% of benign variants are misclassified as deleterious by these tools (44.8% for FATHMM-XF, 50.2% for ClinPred and 50.3% for Eigen). For M-CAP (DOR value < 15), we observed that its inherent hyper-sensitivity design (capacity to correctly categorize deleterious variants) comes at the cost of a poor specificity and consequently 46% of benign variants are misclassified as deleterious. It should be stressed that misclassified variants (benign as deleterious and *vice versa*) will contribute to the low resolution rate of exome analysis and to the generation of extended lists of candidate variants, hence hindering the identification of the one or two “causative” deleterious variants.

Finally, we observed that MISTIC and REVEL both have a balanced sensitivity and specificity, *i*.*e*. a balanced ability to correctly classify both deleterious and benign variants. MISTIC misclassified 11% of the benign variants and 17% of the deleterious variants, while REVEL misclassified 16% of benign variants and 27% of deleterious variants. The same tendency was observed when comparing the distribution of variants without MAF (S3 Fig). This analysis provides further demonstration of the balanced ability of MISTIC to discriminate between deleterious and benign variants in comparison to other tools.

### Effect of MISTIC design on its performance

To understand how the different factors incorporated in the original design of MISTIC (namely the Soft Voting system, the composition of the training set and the confidence in the status of the associated variants) contribute to its high performance and best ranking capacity, we generated different MISTIC models and compared their performance in the Del_EvalSet—Benign_EvalSet and the Del_EvalSet—PopSpe_Evalset scenarios.

Several commonly used individual classification models were trained using the same protocol as previsouly described for MISTIC (S4 Fig). On the Del_EvalSet—Benign_EvalSet, the Random Forest (RF) algorithm was the best model (mean AUC: 0.976 ± 0.004), followed by the Ada Boost (AB) model (mean AUC: 0.973 ± 0.003) and the Logistic Regression (LR) model (mean AUC: 0.954 ± 0.005). However, on the Del_EvalSet—PopSpe_Evalset, the LR model was the best model (mean AUC: 0.959 ± 0.010), followed by the RF model (mean AUC: 0.956 ± 0.014) and the AB model (mean AUC: 0.948 ± 0.017).

We further explored the concordance between these three models on the data from the evaluation scenarios (S5 Fig). Overall, on the Del_EvalSet—Benign_EvalSet data (S5A Fig), there is more than 80% of concordance among all the models (82.86% on benign variants; 89.45% on deleterious variants). The concordance is even higher among the two tree-based approaches (RF and AB models) with a concordance of 92.90% on benign variants and 92.21% on deleterious variants. The major difference between the LR model and the RF model is that it generated 11.62% more false positive deleterious predictions on benign variants with MAF. However, on the Del_EvalSet—PopSpe_EvalSet data (S5B Fig), while there is a 94.92% concordance among all models on deleterious variants, there is a concordance of only 10.68% for benign variants without MAF. The concordance between the tree-based approaches (RF and AB models) is of 99.44% on deleterious variants and 10.68% on benign variants. The AB model generated 29.50% more false positive deleterious predictions on benign variants without MAF. However, the concordance between the RF and the LR models is higher on benign variants without MAF, with a concordance of 40.18%. The LR additionally predicts 39.88% of true negative benign variants, which were mispredicted by the RF model. Hence, to avoid an unbalanced voting system towards false positive predictions on benign variants without MAF, we retained only the RF and the LR models in our Soft Voting system for MISTIC.

To investigate the contribution of the Soft Voting system (based on the weighted average of the RF and the LR models), we compared the full MISTIC model using Soft Voting to the RF and LR models in the different evaluation scenarious (S15 Table). The Soft Voting approach has the most balanced performance on both evaluation sets (Del_EvalSet-Benign_EvalSet: AUC of 0.969 and Del_EvalSet-PopSpe_EvalSet: AUC of 0.962). While the RF approach has the highest sensitivity, its specificity dropped from 0.902 on Benign_EvalSet to 0.466 on PopSpe_EvalSet. As for the LR approach, while it has also a balanced performance, its AUC and DOR values were systematically lower than the Soft Vorting system on both scenarios. On average, over the two evaluation scenarios, the Soft Voting system has an improved performance for F1 score by 4.355 ± 10.932% and specificity by 12.423 ± 31.833%.

To investigate the potential contribution of combining deleterious and benign sets with a wide spectrum of variants in the training set, we compared the full MISTIC model to alternative models using a single source of deleterious variants (ClinVar only or HGMD only) or a source of benign variants with a reduced spectrum of variants in terms of ethnic groups or number of benign variants (UK10K or ClinVar). The full MISTIC model (using a training set of deleterious variants from both ClinVar and HGMD) exhibits a small improvement of AUC (1.223 ± 0.430%) compared to the models using only one source of deleterious variants (S16 Table). The source of benign variants had a greater impact on MISTIC performance (S17 Table), improving the AUC by 3.007 ± 3.026%, the Log Loss by 34.478 ± 22.739%, and the specificity by 34.314 ± 33.585% compared to the models using either UK10K or ClinVar benign variants only. This suggests that, although benign variants from curated databases such as ClinVar can be useful for improving the machine-learning definition of deleterious variants, these databases do not contain the full spectrum of benign variants that are present in population databases. This is also true for the model using benign variants from the UK10K set, which has a partial representation of the diverse ethnic groups.

Finally, to evaluate the impact of the high confidence training set, we compared the full MISTIC model to a model in which no high-confidence filtering criteria was applied (described in material and methods) for ClinVar variants (Pathogenic and Likely Pathogenic CLNSIG status) and HGMD variants (DM and DM? variants). The results in S18 Table show that the full MISTIC model using a high confidence training set increases the F1 score by 3.70 ± 0.97%, the Log Loss value by 31.95 ± 16.84%, and the specificity value by 8.83 ± 1.50%, compared to the model without high confidence filtering.

## Discussion

With the widespread use of exome analyses for the study of rare Mendelian diseases, the major challenge hindering a complete transfer into routine clinical usage remains the interpretation of the list of VUS to identify the one or two “causative” deleterious variants. The list of VUS (mostly composed of missense variants) in unsolved exomes is generally too extensive to be screened manually or *via* experimental assays. Consequently, several tools have been developed to distinguish deleterious and benign variants and hence prioritize candidate variants for further validations assays. However, current solutions implement different strategies and can have large variations in performance.

MISTIC is a prediction tool combining a voting system with two complementary algorithms, which is dedicated specifically to the prediction of deleterious missense variants, in contrast to some generalist prediction tools aimed at predicting different types of variants (coding and noncoding) with diverse consequences (missense, nonsense, splice…). The performance of MISTIC and the other prediction tools were benchmarked on different evalution sets corresponding to diverse variant analysis scenarios ranging from evaluation of novel deleterious variants (ClinVarNew) and variants from different sources (DoCM), to rare benign variants with (Benign_EvalSet) or without (PopSpe_EvalSet) MAF information. Our results show that, in all the different evaluation scenarios, dedicated missense prediction tools (*e*.*g*. ClinPred, REVEL, M-CAP, PrimateAI and MISTIC) perform better than generalist ones (*e*.*g*. Eigen and FATHMM-XF). In this context, MISTIC exhibits the best performance compared to the other dedicated prediction tools. The results were obtained *via* objective analyses using independent evaluation sets (disjoint from the training set) to exclude any type I circularity error and selecting only variants with a score available for all the tools tested. Nevertheless, it is important to note that the training sets of Eigen, ClinPred, M-CAP and REVEL were not readily accessible, and we could not exclude overlapping variants in the evaluation sets. In some cases, this might lead to an over-estimation of the performance for some tools. For instance, this was potentially the case for ClinPred on the DoCM set, where it had a sensitivity value of 1.

The improved performance of MISTIC can be attributed to the special care taken in its design. We evaluated and showed the impact of the different original design elements on MISTIC performance. First, this is, to our knowledge, the first usage of a combination of two different classes of machine-learning algorithms (Random Forest and Logistic Regression). In contrast, the other prediction tools use a single algorithm (Eigen, PrimateAI, FATHMM-XF, REVEL, M-CAP) or two similar ones (ClinPred uses two tree-based algorithms). Furthermore, MISTIC exploits the two machine learning algorithms in a Soft Voting system with optimized hyper parameters after a grid search with 20 iterations and 5 cross-validations. This synergic design results in a balanced sensitivity and specificity ratio (Fig 4, S3 Fig, and S15 Table) and thus a better classification of both deleterious and benign variants.

Second, MISTIC incorporates 113 features out of the initial 714 features, after a selection by Recursive Feature Elimination. This reduced set of features is used to characterize missense variants, ranging from the DNA level with the multi-ethnic MAF and evolutionary constraint features, to the amino-acid level with physiochemical and biochemical property changes. Since our training set is enriched in high confidence deleterious and benign variants, we expected that informative weighted features for distinguishing rare deleterious variants from rare benign variants could be identified. By studying the relative weights of the 113 features used by the two algorithms, we observed that the most predominant features for the Random Forest are the global MAF value and the MetaSVM score, while the MetaLR and VEST4 scores are the most predominant ones for the Logistic Regression. Overall, the integration of these features in two complementary machine algorithms may explain the overall best performance of MISTIC for the discrimination of deleterious variants from benign variants.

The third improvement in MISTIC’s design is the constitution of its positive and negative training sets. The existing missense prediction tools used only one source of deleterious variants for the training of their model, either HGMD Pro (FATHMM-XF, REVEL and M-CAP) or ClinVar (ClinPred, Eigen). We showed that with a positive set composed of a wider spectrum of deleterious variants from multiple sources (ClinVar and HGMD Pro), MISTIC was able to improve its AUC value by 1%, its specificity by 3% and its Log Loss value by 6% (S16 Table). Moreover, to reduce the impact of misclassified deleterious variants, only the highest-confidence deleterious variants (with respect to each source) were used to train MISTIC, while tools like ClinPred also included variants with a ‘likely pathogenic/deleterious’ status in their training set. We showed that the use of a high-confidence positive set in MISTIC had the most impact on performance, increasing the specificity and Log Loss values by 34% (S18 Table). Concerning the negative training set, special attention was also taken to include a wide spectrum of rare benign variants from large control population databases. We also ensured that the negative set was distinct from the positive set, by filtering all the variants already present in the ClinVar and HGMD Pro databases, or other training sets (circularity error) in order to identify informative predictive features for rare benign variants. Our results show that this strategy improved MISTIC’s AUC value by 3% and its specificity by 34% (S17 Table). The same tendency was observed for population specific variants, where other tools (REVEL, M-CAP) trained on negative sets from control population databases performed better than tools trained on a limited set of benign variants (*e*.*g*. ClinPred uses benign variants from ClinVar) (Fig 2C). Taken together, the constitution of a high confidence training set, with sources representing a wider spectrum of variant profiles contributed to the performance of MISTIC in complex scenarios encountered in exome analyses.

The MAF feature, which is part of the ACMG recommendation, has previously been shown to be a powerful factor for filtering benign variants and it is already integrated in the other tools with various strategies. Hence, we construced evaluation scenarios using variants with/without MAF and in both cases we demonstrated that MISTIC had the best performance. MISTIC achieved an AUC improvement of 5% compared to the second-best performing tool on variants with MAF (VarTest) and an AUC improvement of 6% on variants without MAF.

Finally, in a context of routine clinical exome analysis, the major objective is to obtain a limited list of VUS (major challenge in 70% of unsolved exomes) with prioritized candidate variants that can be quickly screened experimentally with reasonable resources. The performance of some prediction tools on the simulated disease exomes (1KG) and real clinical exomes (MyoCapture) was contrasted with the previous evaluation results. Indeed, in the context of an exome analysis, PrimateAI obtained the best performance in terms of the smallest number of VUS (<20%), while M-CAP produced twice as many. However, in terms of ranking the causative deleterious variants, MISTIC achieved the best ranking performance on the simulated disease exomes (P < 1.21E-17) and the same tendency was observed on the real clinical exomes (P < 0.045). Taken together, these results illustrate that the balanced sensitivity and specificity of MISTIC in the different scenarios can also be applied in a context of personalized and precision medicine, in order to obtain a short list of prioritized candidate variants that is amenable to expert screening with reasonable resources.

In conclusion, MISTIC is a novel tool for prediction of deleterious of missense variants, based on a Soft Voting system of two complementary optimized supervised machine-learning algorithms. Among the 113 features integrated in MISTIC, multi-ethnic MAF are predominant for the classification of benign and deleterious variations. MISTIC consistently outperforms recent state-of-the-art prediction tools in the different scenarios tested. Finally, we provide a pre-computed score for all possible human missense variants (for canonical transcripts on the genome version GRCh37) in order to facilitate usage and integration in analysis pipelines. The source code of the method is available on the website http://lbgi.fr/mistic.

Future improvements will include additional informative features, such as multi-ethnic MAF from other population databases, genotype frequencies, and gene-based calibration of the different scores. Moreover, our approach could be applied for the design of dedicated prediction tools for other categories of variants, such as splice variants or non-coding variants, to prepare the transition from exome to complete genome analyses.

## Supporting information

S1 Fig Selection of MISTIC features. The pruning of the initial 714 missense features was performed using the Recursive Feature Elimination method for the Random Forest and Logistic Regression models and the VarTrain set. The red dotted line indicates the cutoff for the selected features in the final Soft Voting system.
(TIF)

S2 Fig Relative normalized weights of the individual missense features integrated in MISTIC. The histograms show the relative weights of the individual missense features for the Random Forest and Logistic Regression models integrated in MISTIC. Key: MAF—minor allele frequency, AFR—African American population, AMR—Latino American population, ASJ–Ashkenazi Jewish population, EAS—East Asian population, FIN—Finnish population, NFE—Non-Finnish European population, SAS–South Asian population, CCRS—Constrained-Coding RegionS.
(TIF)

S3 Fig Distribution of scores for deleterious and benign variants without MAF. The variants of the deleterious (Del_EvalSet) and benign sets (PopSpe_EvalSet) without MAF were pooled and the distribution of the scores for deleterious and benign variants were represented using violin plots. Red area–distribution of scores for deleterious variants. Green area–distribution of scores for benign variants. Black line–recommended threshold.
(TIF)

S4 Fig Performance of classifier models on data from the evaluation scenarios. Different individual classifier models were evaluated for their ability to discriminate deleterious variants from rare benign variants and population-specific missense variants. All classifier models were evaluated using: A—Del_EvalSet-Benign_EvalSet corresponding to novel deleterious variants, known deleterious variants from diverse sources and rare benign variants with MAF data (<0.01, <0.005, <0.001, <0.0001, singleton). B—Del_EvalSet-PopSpe_Evalset corresponding to novel deleterious variants, known deleterious variants from diverse sources and benign variants without MAF data.
(TIF)

S5 Fig Concordance among classification models on data from the evaluation scenarios. Binary predictions made by the 3 classification models for each benign or deleterious variant in the Del_EvalSet, Benign_EvalSet and PopSpe_Evalset scenarios are shown in the upper and lower panels. Each variant is represented by a *row* and a *red or green* tile depicts a deleterious or benign prediction, respectively, by the corresponding classification model. A–Prediction of the classification models for Del_EvalSet–Benign_EvalSet variants (1990 deleterious variants; 1990 benign variants). B–Prediction of the classification models for Del_EvalSet–PopSpe_EvalSet variants (983 deleterious variants; 1062 benign variants).
(TIF)

S1 Table List of missense features. (XLSX)

S2 Table Generation of VarData set for the training and testing of MISTIC. Different filters were applied in order to generate balanced positive set (high-confidence deleterious variants) and negative set (benign variants). a–Selection of variants with a "Pathogenic" information in the clinical significance (CLNSIG) INFO tag in ClinVar VCF file. b–Selection of at least two-stars high-confidence variants with either 'criteria_provided', '_multiple_submitters', 'reviewed_by_expert_panel', 'practice_guideline' or 'no_conflicts' information in the clinical review status (CLNREVSTAT) INFO tag in ClinVar VCF file. c–Selection of high-confidence missense variants with a Disease-Mutation (DM) STATUS INFO tag in HGMD Pro VCF file. d–Selection of missense variants with a depth coverage > 30X and absent from ClinVar and HGMD Pro databases. e–Filtering of variants that overlap any of the training set variants of SIFT, PolyPhen-2, Condel, VEST4, CADD, MetaLR/MetaSVM.
(XLSX)

S3 Table Generation of evaluation sets for the benchmark of prediction tools. N/A: Not Applicable. *: Mean value per exome.
(XLSX)

S4 Table List of recommended thresholds used for the prediction tools. *—There was no recommended threshold for Eigen. Hence, by default the threshold of Eigen was set to 0. N/A: Not Applicable.
(XLSX)

S5 Table List of metrics used to compare the performance of the prediction tools. (XLSX)

S6 Table Selection of features for MISTIC models. The features for the Random Forest and the Logistic Regression models, integrated in the MISTIC Soft Voting system, were selected after applying a Recursive Feature Elimination method.
(XLSX)

S7 Table List of grid-searched optimized hyper-parameters for the Soft Voting systems of MISTIC. These values were obtained after 20 iterations and 5 cross-validations.
(XLSX)

S8 Table Features integrated in the Soft Voting system of MISTIC. The cells highlighted in green correspond to the most important features for each algorithm.
(XLSX)

S9 Table Benchmark metrics of missense prediction tools on VarTest set. Best scores are in bold.
(XLSX)

S10 Table Benchmark of prediction tools on ClinVarNew deleterious missense variants and BenignEvalSet rare benign missense variants. Best scores are in bold.
(XLSX)

S11 Table Benchmark of prediction tools on DoCM deleterious missense variants and BenignEvalSet rare benign missense variants. Best scores are in bold.
(XLSX)

S12 Table Benchmark of prediction tools on deleterious sets of missense variants and population-specific benign missense variants. Best scores are in bold.
(XLSX)

S13 Table Evaluation of missense prediction tools on simulated disease exomes. The p-value results from the statistical test comparing the results of MISTIC to other prediction tools. The best performances are in bold. N/A: Not Applicable.
(XLSX)

S14 Table Evaluation of prediction tools on real clinical exomes from the MyoCapture project. The best performances are in bold.
(XLSX)

S15 Table Evaluation of the contribution of the Soft Voting system in the performance of MISTIC. The best performances are in bold.
(XLSX)

S16 Table Evaluation of the contribution of the source of deleterious variants in the performance of MISTIC. The best performances are in bold.
(XLSX)

S17 Table Evaluation of the contribution of the source of benign variants in the performance of MISTIC. The best performances are in bold.
(XLSX)

S18 Table Evaluation of the impact of the high confidence training set of deleterious variants on the performance of MISTIC. The best performances are in bold.
(XLSX)
