# Supplementary figures and images for: MISTIC: A prediction tool to reveal disease-relevant deleterious missense variants
Source: PLoS One. 2020 Jul 31;15(7):e0236962. doi: 10.1371/journal.pone.0236962 (PMC7394404; doi:10.1371/journal.pone.0236962)

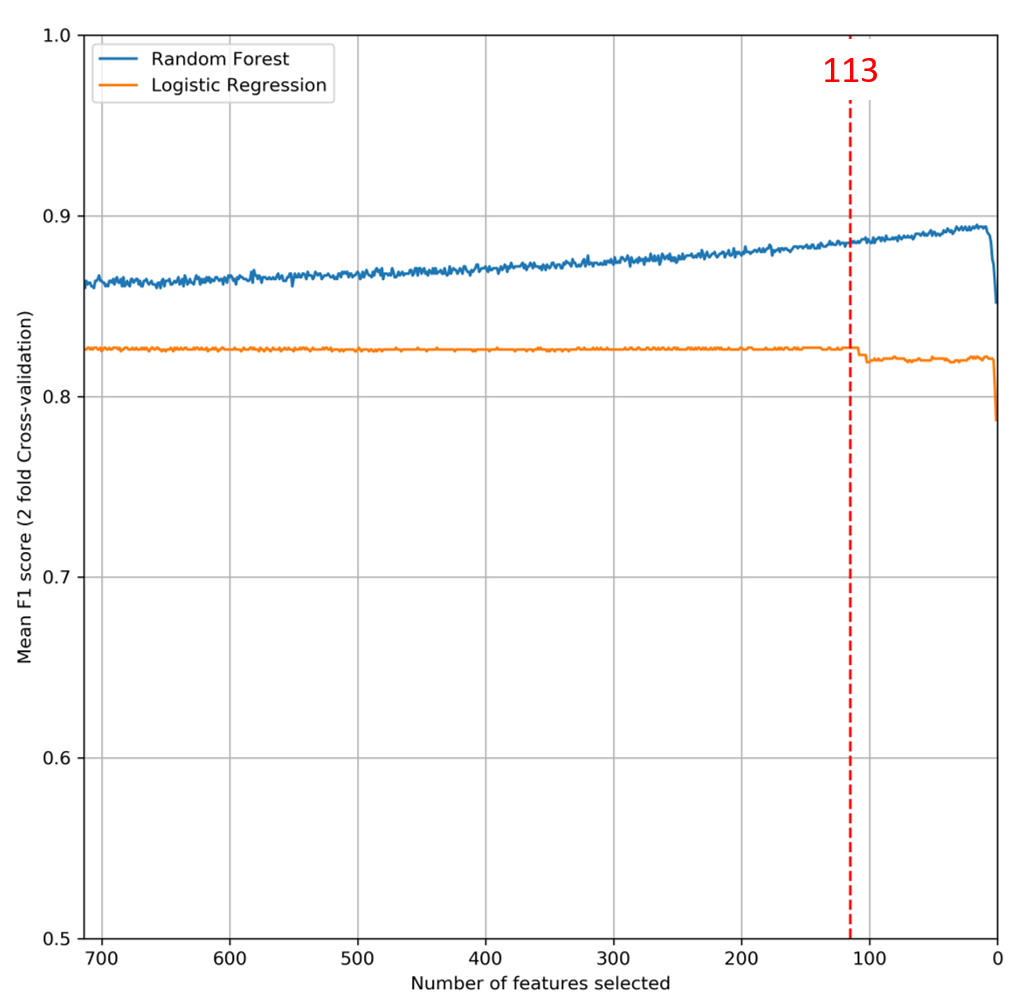

Supplement: S1 Fig — The pruning of the initial 714 missense features was performed using the Recursive Feature Elimination method for the Random Forest and Logistic Regression models and the VarTrain set. The red dotted line indicates the cutoff for the selected features in the final Soft Voting system. (TIF) [file pone.0236962.s001.tif]

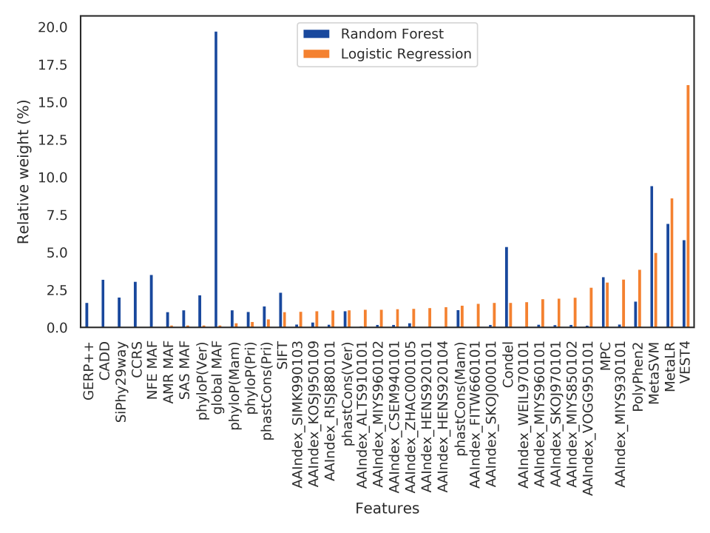

Supplement: S2 Fig — The histograms show the relative weights of the individual missense features for the Random Forest and Logistic Regression models integrated in MISTIC. Key: MAF—minor allele frequency, AFR—African American population, AMR—Latino American population, ASJ–Ashkenazi Jewish population, EAS—East Asian population, FIN—Finnish population, NFE—Non-Finnish European population, SAS–South Asian population, CCRS—Constrained-Coding RegionS. (TIF) [file pone.0236962.s002.tif]

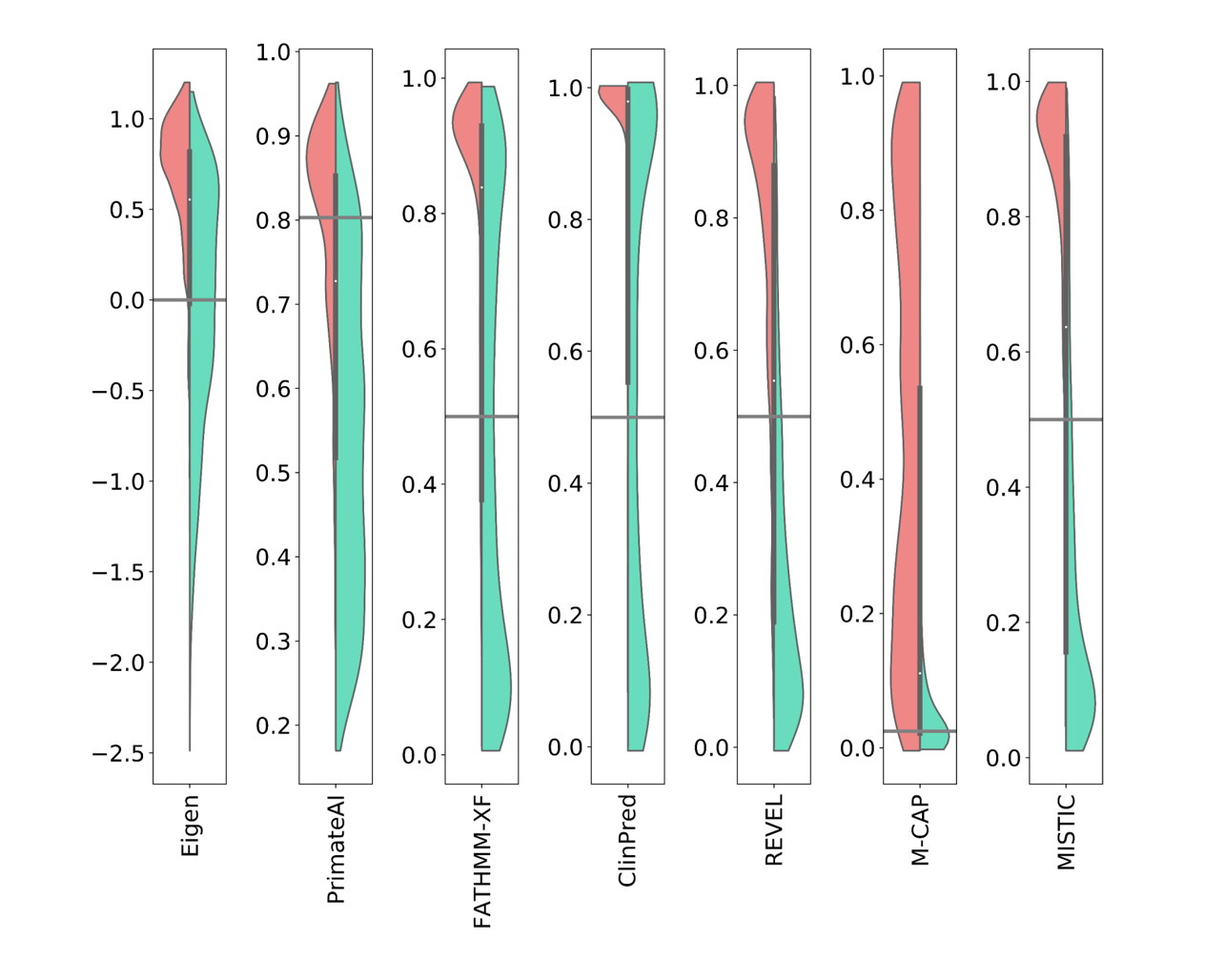

Supplement: S3 Fig — The variants of the deleterious (Del_EvalSet) and benign sets (PopSpe_EvalSet) without MAF were pooled and the distribution of the scores for deleterious and benign variants were represented using violin plots. Red area–distribution of scores for deleterious variants. Green area–distribution of scores for benign variants. Black line–recommended threshold. (TIF) [file pone.0236962.s003.tif]

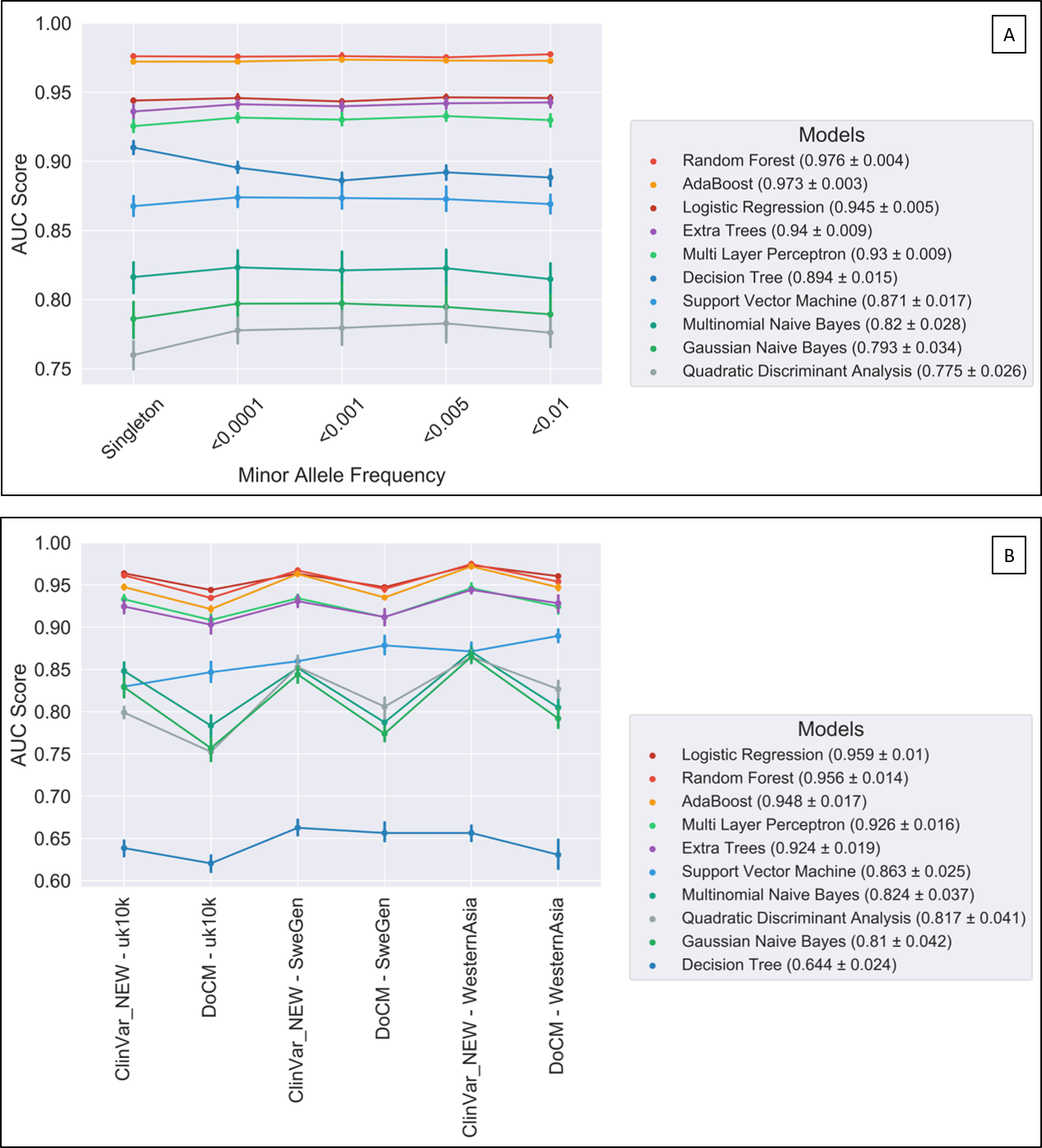

Supplement: S4 Fig — Different individual classifier models were evaluated for their ability to discriminate deleterious variants from rare benign variants and population-specific missense variants. All classifier models were evaluated using: A—Del_EvalSet-Benign_EvalSet corresponding to novel deleterious variants, known deleterious variants from diverse sources and rare benign variants with MAF data (<0.01, <0.005, <0.001, <0.0001, singleton). B—Del_EvalSet-PopSpe_Evalset corresponding to novel deleterious variants, known deleterious variants from diverse sources and benign variants without MAF data. (TIF) [file pone.0236962.s004.tif]

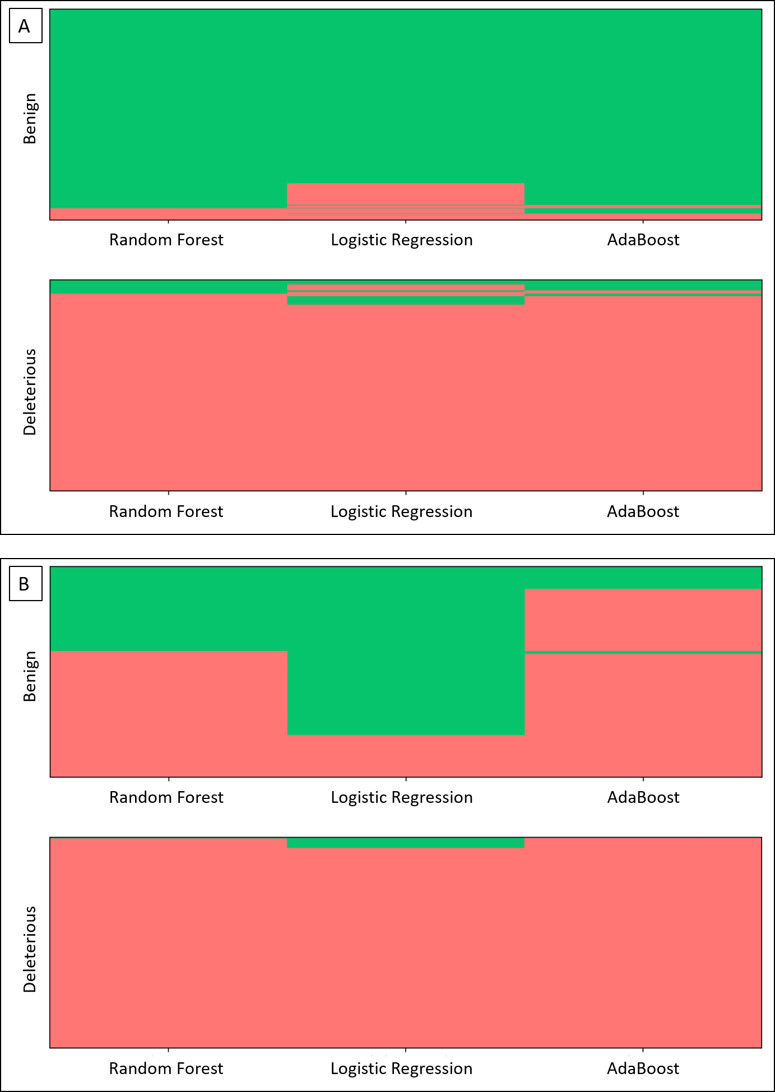

Supplement: S5 Fig — Binary predictions made by the 3 classification models for each benign or deleterious variant in the Del_EvalSet, Benign_EvalSet and PopSpe_Evalset scenarios are shown in the upper and lower panels. Each variant is represented by a row and a red or green tile depicts a deleterious or benign prediction, respectively, by the corresponding classification model. A–Prediction of the classification models for Del_EvalSet–Benign_EvalSet variants (1990 deleterious variants; 1990 benign variants). B–Prediction of the classification models for Del_EvalSet–PopSpe_EvalSet variants (983 deleterious variants; 1062 benign variants). (TIF) [file pone.0236962.s005.tif]
